# Genetic resources and pangenome analysis of barley

**DOI:** 10.1270/jsbbs.24029

**Published:** 2025-02-15

**Authors:** Kazuhiro Sato

**Affiliations:** 1 Faculty of Agriculture, Setsunan University, 45-1 Nagaotoge-cho, Hirakata, Osaka 573-0101, Japan; 2 Kazusa DNA Research Institute, 2-6-7 Kazusa-kamatari, Kisarazu, Chiba 292-0818, Japan

**Keywords:** *Hordeum vulgare*, genome sequencing, genetic resources, genome diversity, crop genebank

## Abstract

Barley (*Hordeum vulgare*) is widely cultivated, ranking fourth in cultivation area among cereal crops worldwide. Many wild and cultivated barley accessions have been collected and preserved in crop genebanks throughout the world. Barley has a large genome (~5 Gbp) that has recently been sequenced and assembled at the chromosome level by the international research community. The community also is sequencing accessions representing the diversity of both domesticated and wild barley to provide genome-wide genotyping information for pangenome analysis. Given that the pangenome represents the universe of genome sequences existing in a species, the long-term goal of this project is to obtain high-quality genome sequences of the major barley accessions worldwide. As each accession is annotated, the capacity to explore structural differences is enhanced by the increased understanding of the diversity of the barley genome, which will facilitate efficient development of cultivars for human consumption. This review describes our current knowledge of barley genome diversity and proposes future directions for basic and applied research of the barley pangenome.

## Sources of genome diversity in barley

Barley (*Hordeum vulgare* L.) is a self-pollinating crop with a diploid genome consisting of seven chromosomes (2*n* = 2*x* = 14). The estimated genome size of barley is 5.10 Gbp (IBSC 2012), comprising over 80% repetitive elements. Among Poaceae species, the genetics of barley has been particularly well studied. In addition, several mutant traits have practical importance and can be used as model traits in cereal crops. Notably, many barley and wheat (*Triticum aestivum*) genes have similar functions. Therefore, information about a gene in barley can be readily applied to identify the genes responsible for a similar trait in wheat, as demonstrated by the genome editing of a triple-knockout mutant of the wheat gene *Quantitative trait locus seed dormancy 1* (*Qsd1*) (Abe *et al.* 2019), which was originally identified in barley (Sato *et al.* 2016a).

**Fig. 1.**
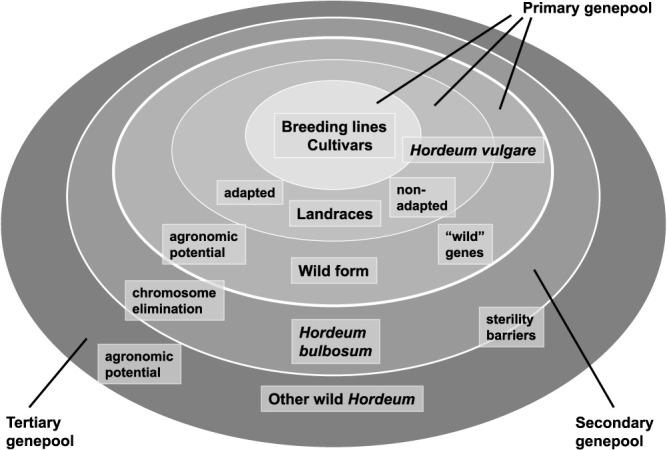
Gene pools of cultivated barley (*Hordeum vulgare* ssp. *vulgare*) (Bothmer *et al.* 2003).

The genus *Hordeum* comprises over 30 species, including cultivated barley (*Hordeum vulgare* ssp. *vulgare*) (Blattner 2009). Of the gene pools listed in Fig. 1, only the ancestral form of cultivated barley, *H*. *vulgare* ssp. *spontaneum*, belongs to the primary gene pool of cultivated barley. This ancestral form of wild barley shares the same genome as domesticated barley and is classified as a barley subspecies (Bothmer *et al.* 2003). Crosses of cultivated barley with *H*. *vulgare* ssp. *spontaneum* show no incompatibility barriers; hence, there is a full capacity for gene transfer.

*H. vulgare* shares the basic genome H with *H. bulbosum*, which is considered to be the only species in the secondary gene pool (Fig. 1). *H. bulbosum* has been used for crossing with cultivated barley to produce hybrids or for haploid induction due to chromosome elimination of *H. bulbosum* in hybrid progeny. Other *Hordeum* species are less closely related to cultivated barley and are classified as the tertiary gene pool of cultivated barley (Fig. 1). Most of these species share variants of the basic genome I, whereas two widespread weeds, *H. marinum* and *H. murinum*, possess distinct genomes (Xa and Xu, respectively; Bothmer *et al.* 1995).

Before barley was domesticated, the ancestral wild form of barley (*H. vulgare* ssp. *spontaneum*) was used as a human food, possibly to make an early form of flatbread using grains milled with stone tools, and the dough was baked in an open fire. Both wild and domesticated barley have been found in archaeological sites in the Fertile Crescent, which is considered to be the origin of barley domestication, dating back ca. 10,000 years (Zohary *et al.* 2012). As described by Sato (2020), wild barley kernels can be distinguished from domesticated barley kernels by their brittle and smooth rachis, which contrasts with the non-brittle and tough rachis of domesticated barley (Pourkheirandish *et al.* 2015). Millions of generations of this wild barley provide a source of diversity to the present-day cultivated form, although domestication partially narrowed the diversity of barley. Soon after its domestication, mutations responsible for agronomically valuable traits, e.g., six-rowed spikes (Komatsuda *et al.* 2007) and hull-less caryopses (Taketa *et al.* 2008), have been selected.

Allele diversity at agriculturally important loci associated with diverse ecological conditions and the different uses of this cereal (human food, animal feed, and malt production) have made barley landraces well suited for cultivation throughout the world. Naturally occurring diversity in locally adapted landraces and cultivars developed from landraces remains the most common source of available diversity for the crossbreeding of barley (Fischbeck 2003). Beyond crossbreeding, research on induced mutations to improve barley was initiated in the 1920s. Great efforts were made to increase the frequency of useful mutations and to control the process (Lundqvist and Franckowiak 2003). The numerous barley mutants produced by these efforts have greatly facilitated basic research and have contributed to practical breeding programs.

## Collection of genetic resources in barley

Barley germplasm has been extensively collected owing to the economic importance of this crop for agricultural production and for malting and producing animal feed (Ullrich 2011). Thus, most germplasm collections are composed of landraces of *Hordeum vulgare* ssp. *vulgare*. According to Knüpffer (2009), there are over 485,000 accessions of *Hordeum* residing in more than 200 genebanks worldwide. These collections include 299,165 accessions of *H*. *vulgare* ssp. *vulgare*, 32,385 accessions of *H. vulgare* ssp. *spontaneum*, and 4,681 accessions of other wild *Hordeum* species. From these accessions, an international barley core collection consisting of approximately 1,500 accessions was developed (Knüpffer and Hintum 2003).

## Estimating genomic variation in barley

Genotyping entire barley genebank collections generates comprehensive information about the genetic diversity and population structure within the accessions and provides efficient tools for barley breeding. The long-term goal is to manage and maintain collections of plant genetic resources by integrating biological and digital sequence information to enhance crop diversity and breeding. The first step is to collect genome-wide genotyping data for all accessions in a genebank collection. Several platforms can be used for genome-wide genotyping of barley. The Illumina iSelect single nucleotide polymorphism (SNP) array (Bayer *et al.* 2017) is an easy choice, but it may introduce ascertainment bias (Moragues *et al.* 2010) due to the pre-selected germplasm used to identify SNPs. Exome capture sequencing and whole-genome shotgun analysis require the construction of libraries, which prohibits the analysis of a large number of samples using complexity reduction and parallel sample processing.

Researchers at the Leibniz Institute of Plant Genetics and Crop Plant Research (IPK) in Gatersleben, Germany used genotyping-by-sequencing (GBS) to collect genotypic data for all ~22,000 barley accessions collected in the German federal genebank (Milner *et al.* 2019). GBS involves digesting DNA with two different restriction enzymes (e.g., *Pst*I and *Msp*I) prior to high-throughput sequencing (Poland *et al.* 2012). GBS does not require prior knowledge of target sequences or pre-analysis to design markers. GBS is a highly streamlined, multiplexed wet-lab protocol that relies only on standard laboratory techniques and reagents (Elshire *et al.* 2011). Although more powerful marker-based platforms may be available in the future, GBS is becoming a major platform for genotyping international barley collections in genebanks worldwide.

## History of analysis of the barley genome

### Draft genome sequencing

The International Barley Genome Sequencing Consortium (IBSC) was established in 2006 to generate a high-quality barley genome sequence using a combination of sequencing mapped gene-bearing BAC clones (BAC-by-BAC strategy) and whole-genome shotgun sequencing (IBSC 2012). From the 5.10 Gbp genome of American malting barley cultivar ‘Morex’, IBSC developed a physical map for 4.98 Gbp, with more than 3.90 Gbp anchored to a high-resolution genetic map, including 26,159 high-confidence genes with cDNA sequences and homology support from other plant genomes. More than 80% of the genome is occupied by repeat sequences.

### Chromosome-scale assembly of barley

For chromosome-scale, high-quality assembly of the barley genome, each BAC clone from the minimum tiling path was barcoded and sequenced using Illumina short reads. A high-resolution genetic map and a highly contiguous optical map (Irys system, BioNano Genomics Inc.) were then combined to construct super-scaffolds composed of merged assemblies from individual BACs. Finally, chromosome conformation capture sequencing (Hi-C) was used to order and orient the BAC-based super-scaffolds. The final chromosome-scale assembly represents 4.79 Gbp (~95%) of the genome of the ‘Morex’ reference assembly (MorexV1) (Mascher *et al.* 2017). Mapping of transcriptome data identified 39,734 high-confidence loci and 41,949 low-confidence loci based on sequence similarity to related species.

### Assembly pipeline

When the IBSC planned the BAC-by-BAC strategy for genome assembly (IBSC 2012), whole-genome shotgun assembly of the barley genome using Illumina short reads (150–300 bp) was performed as a supplementary technique, since less than half of the total genome was assembled owing to the failure of redundant repeat sequences to assemble properly (Sato *et al.* 2016b). However, chromosome-scale assembly using only short reads became available after the release of NRGene’s commercial genome assembly service DeNovoMAGIC.

The open-source assembly pipeline TRITEX (Monat *et al.* 2019) has also been developed. The purpose of the TRITEX pipeline is to construct chromosome-scale sequence assemblies for Triticeae genomes from different Illumina sequencing datasets within several weeks. The target applications of this technique include pangenomic studies in barley and wheat. The TRITEX pipeline only works with Illumina sequencing data of sufficient coverage and for a certain set of libraries. Paired-end and mate-pair datasets suitable for use with the pipeline are required, together with chromosome conformation capture sequencing (Hi-C and Dovetail Omni-C) data suitable for use with the pipeline and chromium data from 10X Genomics. The TRITEX pipeline must be run on a powerful Unix server with more than 1 TB of RAM. The pipeline requires a few weeks to assemble short-read data from barley and other plants with large genomes (such as wheat), which limits the number of accessions used for pangenome analysis.

### Second version of the reference genome assembly of ‘Morex’

**Fig. 2.**
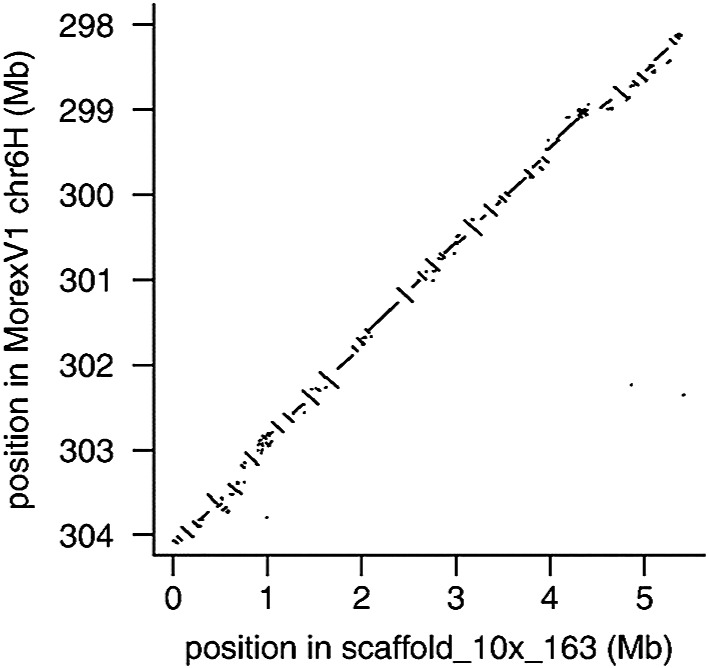
Alignment of MorexV2 assembly scaffold_10x_163 to the MorexV1 assembly (Monat *et al.* 2019).

The TRITEX pipeline was used to construct the second version of the reference genome assembly of ‘Morex’. This MorexV2 assembly resolves the problems of BAC-based reference sequences (Mascher *et al.* 2017) including (1) large sequence gaps, (2) redundancies, and (3) local mis-assemblies (Monat *et al.* 2019). The assembly metrics of the TRITEX MorexV2 assembly greatly exceed those of the BAC-by-BAC MorexV1 assembly (Fig. 2). The proportion of completely aligned full-length (FL)-cDNAs in TRITEX MorexV2 (Matsumoto *et al.* 2011) increased by ~9% compared to the BAC-by-BAC assembly.

## Pangenome analysis of barley

No single genome can represent the genetic diversity of a crop species. The concept of a ‘pangenome’ refers to the universe of genome sequences existing in a species (Jayakodi *et al.* 2021). Analysis of sequence variants segregating in multiple high-quality genome sequences is the goal of pangenome studies. Pangenomic methods have rapidly progressed over the past few years, and it is now practical to propose that common genomic analyses use a pangenome (Liao *et al.* 2023).

**Fig. 3.**
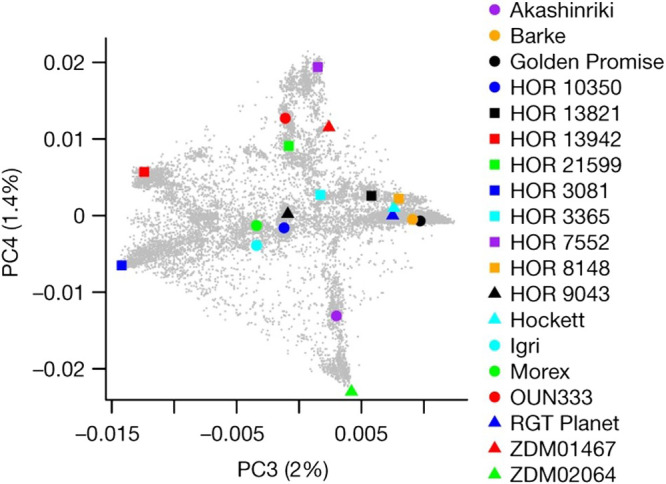
Nineteen domesticated barley genotypes representing the genetic diversity space, as revealed by principal component analysis (PCA) of genotyping-by-sequencing data from 19,778 domesticated barley varieties (Jayakodi *et al.* 2020).

The starting point for pangenomics in barley was a comprehensive survey of species-wide diversity based on genome-wide genotyping of more than 22,000 barley accessions, mainly from the German federal genebank (Milner *et al.* 2019). To obtain a good representation of the primary barley gene pools, the authors selected accessions that were well separated by principal component analysis of GBS data on barley accessions (Fig. 3). In addition to these 18 gene pool representatives, these accessions included the reference genotype ‘Morex’ and one wild barley (*H. vulgare* ssp. *spontaneum*) accession from Israel ‘B1K-04-12’.

### Assembly of 20 barley genotypes

The selected genotypes were assembled using the TRITEX assembly pipeline (*n* = 16), DeNovoMAGIC from NRGene (*n* = 3), or W2rap (https://doi.org/10.1101/110999) (*n* = 1). A comparison of the short-read assembly of ‘Morex’ to a long-read assembly of this genotype generated from PacBio long reads was performed to demonstrate that short-read assemblies are amenable to pangenomic analysis in barley. Although the assemblies of the 20 diverse accessions differed in terms of contiguity and the extent of gap sequences in the intergenic space, they had a similar representation of reference gene models (MorexV2) and were highly co-linear at the whole-chromosome scale (Jayakodi *et al.* 2020).

### Annotation of the barley genome assemblies

A similar proportion (~80%) of the assembled sequence of each genotype was composed of transposable elements (Jayakodi *et al.* 2020). *De novo* gene annotation using Illumina RNA-Seq and PacBio Iso-Seq data was performed for three genotypes: ‘Morex’, ‘Barke’, and the Ethiopian landrace ‘HOR 10350’. Gene models defined based on these three assemblies were consolidated and projected onto 17 other assemblies (gene projection). Between 35,859 and 40,044 gene models in each assembly were annotated by gene projection. The clustering of orthologous gene models yielded 40,176 orthologous groups. Of these, 21,992 occurred as a single copy in all 20 assemblies, 3,236 occurred in multiple copies in at least one of the 20 assemblies, 13,188 were absent from at least one assembly, and 1,760 were present in only one assembly (Jayakodi *et al.* 2020).

### Structural variations in the 20 barley genome assemblies

Multiple genome assemblies from representative accessions are a source of structural variants, which can be used to genotype other genebank accessions with low-coverage sequence data or genome-wide genotyping data. Using Assemblytics (Nattestad and Schatz 2016) software, Jayakodi *et al.* (2020) discovered presence–absence variation (PAV) by performing a pair-wise comparison of 19 chromosome-scale assemblies to the ‘Morex’ reference assembly. The authors identified 1,586,262 PAVs, ranging in size from 50 to 999,568 bp. PAV density was higher in distal, gene-rich regions with higher recombination rates.

Another method for estimating structural variations is to identify single-copy regions extracted from each of the 20 assemblies and cluster them into a non-redundant set of sequences (Jayakodi *et al.* 2020). The average cumulative size of a single-copy sequence in each accession was 478 Mb (ca. 9.5% of the genome assembly). The total size of non-redundant single-copy sequences was 638.6 Mb, including 402.5 Mb of single-copy sequences shared among all 20 genotypes and 235.9 Mb of variable sequences. On average, each of the 20 genotypes contained 2.9 Mb of single-copy sequences not present in any other assembly.

**Fig. 4.**
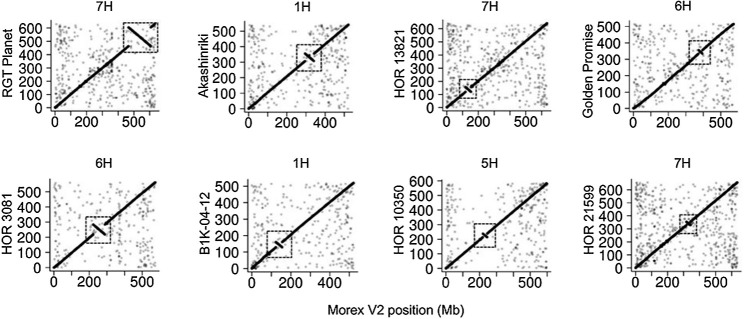
Whole-genome alignments showing some examples of large chromosomal inversions identified using Hi-C data (Jayakodi *et al.* 2020).

There are some chromosome inversions between these assemblies and MorexV2 identified using Hi-C data. These are confirmed as real inversions and not from mis-assemblies. Some of these inversions on the long arms of chromosomes are shown in Fig. 4.

**Fig. 5.**
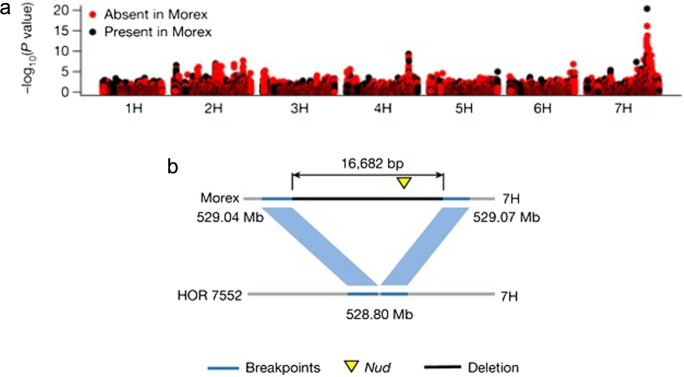
a, Genome-wide association scan for lemma adherence on the basis of present and absent variation (PAV) markers with Morex and other 19 pangenome assemblies. The black and red dots in the Manhattan plot denote single-copy sequences that are present and absent in Morex, respectively. b, The most highly associated PAV marker was a 16.7-kb region that is deleted in the naked accession HOR 7552 and that contains the *NUD* gene (Jayakodi *et al.* 2020).

Based on the analysis of single-copy sequences, Jayakodi *et al.* (2020) selected PAV markers in Morex. The black and red dots in the Manhattan plot in Fig. 5a denote single-copy sequences that are present and absent in Morex, respectively. To test the suitability of the single-copy pangenome for genetic analysis in a wider diversity panel without high-quality genome sequences, Jayakodi *et al.* (2020) collected whole-genome shotgun data (threefold coverage) for 200 domesticated and 100 wild accessions of barley. In genome-wide association scans, the pangenome marker that was most highly associated with lemma adherence covered the *NUDUM* (*NUD*) gene (Taketa *et al.* 2008) (Fig. 5a). All varieties of naked barley—in which lemmas can be easily separated from grains—are thought to trace back to a single mutational event, deleting the entire *NUD* sequence. The most highly associated PAV marker was a 16.7-kb region that is deleted in the naked accession ‘HOR 7552’ and that contains the *NUD* gene (Fig. 5b).

### Additional assemblies

Using a similar strategy to that of Jayakodi *et al.* (2020), two additional assemblies were established, including assemblies of wild accession ‘OUH602’ (Sato *et al.* 2021a) and barley cultivar ‘Haruna Nijo’ (Sakkour *et al.* 2022). These germplasms are key genetic and genomic resources at Okayama University that have been used as parents of mapping populations, genotypes for cDNA analysis, and BAC library development. The authors used the TRITEX pipeline and annotation methodologies from pangenome analysis for chromosome-scale assembly of the two genotypes. Assembly information and browsers are available at the NBRP barley website (http://earth.nig.ac.jp/~dclust/cgi-bin/index.cgi). The FL-cDNA sequences of ‘Haruna Nijo’ (Matsumoto *et al.* 2011, Sato *et al.* 2009) were mapped onto the assembly, which could serve as a key resource for gene identification in barley.

## Recent advancements and future directions

### Long-read assembly

**Fig. 6.**
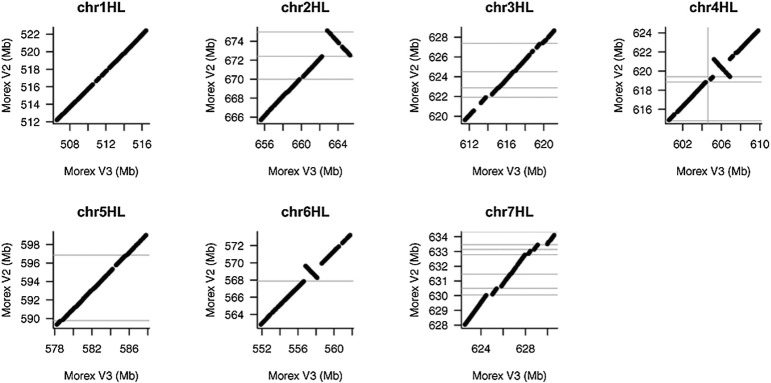
Alignments of MorexV3 and MorexV2 pseudomolecules in the terminal 10 Mb of the long arm of each chromosome. Gray lines indicate scaffold boundaries (Mascher *et al.* 2021).

The recent development of long-read sequencing by circular consensus sequencing (CCS) on the PacBio platform alters the strategy of plant pangenome analysis. Mascher *et al.* (2021) compared the ability of current long-read sequencing platforms to rapidly generate contiguous sequence assemblies in barley (Fig. 6). Long-read assemblies are clearly superior to short-read assemblies in barley. A downsampling study indicated that 20-fold genome coverage of PacBio HiFi reads provide qualified assemblies in barley. A similar downsampling study in hexaploid wheat cultivar ‘Fielder’ by Sato *et al.* (2021b) indicated that 16-fold reads are sufficient for genome assembly in hexaploid wheat, but that 12-fold reads provide most of the information needed for genome assembly. Long reads can be used to construct accurate and complete assemblies of multiple accessions of a species to build pangenome infrastructures in Triticeae crops.

### Telomere-to-telomere assembly

The generation of gapless, telomere-to-telomere (T2T) sequence assemblies of plant chromosomes was first reported in *Arabidopsis thaliana* (Naish *et al.* 2021). T2T assemblies of plant genomes were subsequently reported in various plant species, including crops. Navrátilová *et al.* (2022) used the same strategy employed by plant T2T projects to analyze sequence gaps in the most updated reference genome sequence of MorexV3. They used long-read sequencing data for MorexV3 and ChIP-seq data for the centromeric histone variant CENH3 to estimate the abundance of centromeric DNA, ribosomal DNA, and subtelomeric repeats in the barley genome. However, they could not assemble the T2T sequence in barley since almost all centromeric sequences and 45S ribosomal DNA repeat arrays were absent from the MorexV3 pseudomolecules and that the majority of sequence gaps could be attributed to assembly breakdown in long stretches of satellite repeats.

There is also a gap between the estimated genome size of the ‘Morex’ draft genome (5.10 Gbp; IBSC 2012), the size (4.79 Gbp) of the MorexV1 assembly (Mascher *et al.* 2017), and the 4.0- to 4.5-Gbp genome sizes identified by pangenome analysis using the TRITEX pipeline (Jayakodi *et al.* 2020). The estimated genome sizes of the newer TRITEX MorexV2 assembly and the long-read MorexV3 assembly (both 4.2 Gbp) are not any longer than the initial estimates from the BAC-based physical map.

### Pan-transcriptomes

A pan-transcriptome describes the transcriptional and post-transcriptional consequences of genome diversity from multiple individuals within a species (Waugh *et al.* unpublished). In barley, deep sequencing of the transcriptome (RNA-seq) from ‘Morex’ and FL-cDNAs from ‘Haruna Nijo’ facilitated the annotation of the reference genome of ‘Morex’. The recent development of a single-molecule sequencing technique may also support the sequencing of long transcripts. An international collaborative project aimed at full-transcript sequencing of 20 pangenome genotypes is currently underway (Waugh *et al.* unpublished). These long-transcript sequences will also contribute to the annotation of each assembly in pangenome analysis. As it is essential to isolate intact mature transcripts (mRNA) to obtain FL-cDNA sequences, techniques are being developed to maintain transcript integrity.

### Genomic information and browsers

For genome assembly and annotation, Ensembl Plants provides easy access to the most updated barley genome assembly (MorexV3), including the sequences of chromosomes, genes, transcripts, and predicted proteins. The same website also supports BLAST searches against the barley genome. Similarly, IPK allows users to conduct BLAST searches against all sequence resources published by the worldwide barley community, including activities related to the International Barley Sequencing Consortium. Pangenome graphs will be needed to replace the current single-genotype genome browser, as demonstrated for the human pangenome project. However, the resolution of sequence variation in barley is currently limited to 50 bp (Bayer *et al.* personal communication), and the use of multiple haplotype browsers is limited at present.

## Application of pangenome information in barley

### Toward a digital genebank system in barley

Genebank genomics forms the basis for the digital genebank system (Mascher *et al.* 2019). The ultimate goal of a digital genebank system is the *de novo* sequencing of all natural and engineered germplasm. Preparing a high-molecular-weight DNA sample for library construction for long-read sequencing runs currently takes a few weeks. The assembly (e.g., TRITEX) pipeline can assemble chromosome-scale sequences for a single barley accession within 3 to 4 weeks. A parallel sequencing and computational analysis strategy might save time, but it is not feasible to sequence thousands of accessions by high-quality chromosome-scale assembly using the current technical standards. An automatic gene annotation system must be developed, since the annotation process currently relies on slow, manual data curation steps. For these reasons, the combination of genotyping all genebank accessions and increasing the number of chromosome-scale assemblies of accessions representing barley diversity is a reasonable solution for the near future.

### Contribution of pangenome information to breeding

Natural and induced variation provides opportunities to analyze traits of interest. SNP haplotype–based characterization of genebank accessions and breeding germplasm is the current approach to standardize the materials used for plant breeding. High-quality genome sequences are essential for the basis of haplotype analyses and establishing worldwide digital genebank networks. Association genetics is a suitable way to utilize pangenomes to combine barley sequence variation with phenotypic variation via user-friendly pangenome browsers (Jayakodi *et al.* 2021).

Agronomically important traits are often controlled by multiple interacting genes, which demands deep knowledge of trait-based genetics. To better understand the contribution of genomic sequence variation to various traits, standardized phenotyping is also emerging as an essential goal. Systematic gene modification by genome editing may also provide a functional view of candidate genes of interest in multiple genotypes. Using recently updated sequencing techniques, it is also feasible to include secondary and tertiary gene pools of barley to explore the diversity of the genus *Hordeum*.

Finally, the combined information collected from genomic sequences and the systematic functional analysis of genes provides novel strategies for trait improvement via the sequence-based breeding of barley.

## Author Contribution Statement

K.S. wrote the manuscript.
